# Soluble CD14 Levels Predict Liver Fibrosis in Metabolic Dysfunction-Associated Steatotic Liver Disease (MASLD) Independently of Obesity and Type 2 Diabetes

**DOI:** 10.3390/ijms27073050

**Published:** 2026-03-27

**Authors:** Ilaria Barchetta, Flavia Agata Cimini, Federica Sentinelli, Sara Dule, Valentina Frattina, Giulia Passarella, Maria Neve Hirsch, Alessandro Oldani, Marco Giorgio Baroni, Maria Gisella Cavallo

**Affiliations:** 1 Department of Experimental Medicine, Sapienza University of Rome, 00161 Rome, Italy; ilaria.barchetta@uniroma1.it (I.B.); sara.dule@uniroma1.it (S.D.); valentina.frattina@uniroma1.it (V.F.); giulia.passarella@uniroma1.it (G.P.); marianeve.hirsch@gmail.com (M.N.H.); alessandro.oldani@uniroma1.it (A.O.); gisella.cavallo@uniroma1.it (M.G.C.); 2 Department of Clinical Medicine, Public Health, Life and Environmental Sciences (MeSVA), University of L’Aquila, 67100 L’Aquila, Italy; federica.sentinelli@univaq.it (F.S.); marcogiorgio.baroni@univaq.it (M.G.B.); 3 Neuroendocrinology and Metabolic Diseases, IRCCS Neuromed, 86077 Pozzilli, Italy

**Keywords:** MASLD, soluble CD14, gut–liver axis, liver fibrosis, lipopolysaccharide-binding protein, intestinal permeability, endotoxemia, non-invasive biomarkers, innate immune activation, metabolic disease

## Abstract

Increased intestinal permeability has been implicated in metabolic dysfunction-associated steatotic liver disease (MASLD), but its relationship with liver fibrosis independent of metabolic risk factors remains unclear. The aim of this study was to investigate the relationship between markers of gut-derived immune activation and liver fibrosis in individuals with metabolic disease. We enrolled 139 adults (48.8 ± 11 years; BMI 33.7 ± 9.5 kg/m^2^; 50% type 2 diabetes); liver steatosis and fibrosis were estimated using the Hepatic Steatosis Index (HSI) and Fibrotic NASH Index (FNI); liver biopsies were available in a bariatric subgroup. Plasma soluble CD14 (sCD14) and lipopolysaccharide-binding protein (LBP) levels were measured by ELISA kits, and the LBP/sCD14 ratio was calculated. MASLD was present in 78% of participants; in these individuals, sCD14 levels correlated with HSI and FNI (both *p* < 0.01). In multivariable analysis adjusting for age, sex, BMI, waist circumference, and type 2 diabetes, sCD14 was independently associated with advanced fibrosis (OR: 3.16, 95% CI 1.32–7.55; *p* = 0.010). This association was confirmed by histology (*p* = 0.02). Overall, these findings point to a link between gut-derived immune activation and fibrotic burden in MASLD and provide insight into the pathophysiological relevance of the gut–liver axis in metabolic disease.

## 1. Introduction

Metabolic dysfunction-associated steatotic liver disease (MASLD) is the hepatic hallmark of the insulin resistance-related diseases, and nowadays it represents the main cause of chronic liver disease worldwide. Although the presence of excessive lipid accumulation in the hepatocytes defines the disease, liver fibrosis is the strongest predictor of liver-related complications and overall mortality, independently of obesity and type 2 diabetes (T2D). Large cohort studies and meta-analyses have consistently shown that fibrosis stage, rather than steatosis or necro-inflammatory activity, drives long-term clinical outcomes in MASLD, highlighting the need for early identification of patients at increased fibrotic risk [1,2].

Beyond traditional metabolic risk factors, chronic low-grade inflammation has emerged as a central mechanism in MASLD progression. In this context, increasing attention has been directed toward the gut–liver axis [3]. Impaired intestinal barrier function, commonly referred to as increased gut permeability, facilitates the translocation of bacterial products directly into the portal circulation, promoting hepatic innate immune activation [4]. The lipopolysaccharides (LPS), derived from Gram-negative bacteria, activate inflammatory signaling pathways through the link with its co-receptor CD14 and the formation of the CD14– Toll-like receptor 4 (TLR4) complex, leading to cytokine release, hepatic stellate cell activation, and fibrogenesis [5,6]. Experimental and clinical studies support a pathogenic role of gut-derived inflammatory signals in metabolic liver disease [7,8].

Alterations in gut permeability have been consistently described in obesity and T2D, conditions that frequently coexist with MASLD [9]. However, the clinical relevance of gut-derived inflammatory activation in liver disease progression remains controversial. Several human studies have demonstrated that markers of endotoxemia and gut barrier dysfunction are strongly influenced by adiposity, insulin resistance, and glycemic status, making it difficult to discern whether they reflect metabolic burden or liver-specific pathogenic processes [10,11]. As a result, the identification of biomarkers capturing immune-inflammatory pathways independently of metabolic confounders remains an unmet clinical need.

Soluble CD14 (sCD14) is the circulating form of CD14 and is released by monocytes and macrophages upon endotoxin exposure and immune activation [12]. Another source of sCD14 is the liver, as it is secreted by hepatocytes in response to interleukin-6 (IL-6), being thus considered an acute-phase reactant [13]. Indeed, unlike markers that primarily reflect circulating endotoxin burden, such as the LPS and the LPS-binding protein (LBP), sCD14 mirrors the host innate immune response to gut-derived bacterial products [14]. Thus, it has been proposed as an integrated marker of immune-inflammatory activation related to impaired intestinal barrier function. Elevated sCD14 levels have been reported in metabolic and inflammatory conditions and linked to adverse outcomes in cardiometabolic disease [15,16,17]. However, data on the role of sCD14 in MASLD are scarce. In particular, evidence so far lacked comprehensive metabolic phenotyping and did not systematically address whether the observed associations were independent of obesity and T2D [18]. Consequently, it remains unclear whether sCD14 reflects liver disease severity per se or captures the underlying metabolic milieu.

Therefore, the present study aimed to investigate the association between circulating sCD14 levels and the presence and severity of MASLD, with a focus on liver fibrosis, in relation to obesity and/or T2D.

## 2. Results

A total of 139 individuals were included in the study, comprising 60 men and 79 women, with a mean age of 48.8 ± 11 years and a mean BMI of 33.7 ± 9.5 kg/m^2^. Obesity was present in 57% of participants, while T2D was diagnosed in approximately 50%.

**Figure 1:**
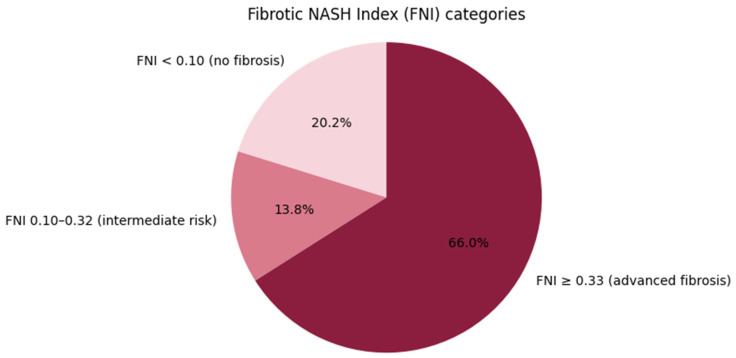
Distribution of liver fibrosis risk categories according to the Fibrotic NASH Index (FNI). *The pie chart shows the proportion of study participants classified into low (<0.10), intermediate (0.10–0.32), and high (≥0.33) fibrosis risk categories based on FNI cut-off values*.

In our study population, 108 out of 139 individuals (78%) met the criteria for MASLD; based on the FNI, approximately two-thirds of the study population showed an increased risk of severe liver fibrosis (FNI > 0.33; Figure 1). Clinical characteristics of the study participants are detailed in Table 1.

When participants were stratified according to FNI-defined liver fibrosis risk, individuals at the highest fibrosis risk (FNI ≥ 0.33) showed a worse metabolic profile compared with those at low–intermediate risk. Despite similar age, sex distribution, BMI, waist circumference, and prevalence of obesity, the high-risk group exhibited significantly lower HDL cholesterol levels and higher triglycerides, fasting plasma glucose, and HbA1c values. Consistently, the prevalence of type 2 diabetes was markedly higher in individuals with advanced fibrosis risk (92% vs. 57%, *p* = 0.001).

Plasma sCD14 concentrations were significantly higher in individuals at high fibrosis risk compared to those at low-intermediate risk (1429.4 ± 373.6 vs. 1224.7 ± 342.6 ng/mL, *p* = 0.023); no significant differences were observed when comparing other markers of endotoxemia, inflammation, and insulin resistance, such as LBP, LBP/CD14, zonulin, TNF-a, and C-RP, as reported in Table 2.

**Figure 2:**
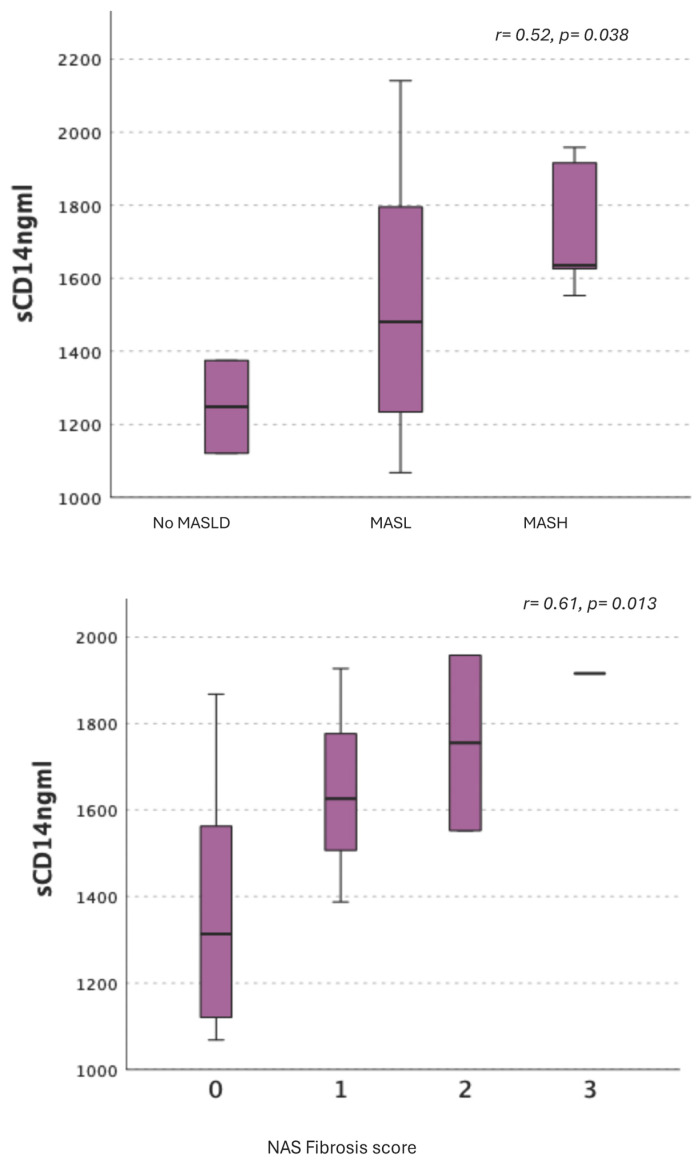
Association between circulating sCD14 levels (ng/mL) and histological diagnosis of MASLD/MASH and fibrosis stage. *Box-and-whisker plots showing plasma sCD14 concentrations (ng/mL). The central line represents the median, boxes indicate the interquartile range (IQR), and whiskers denote minimum and maximum values. **Upper panel**: sCD14 levels according to histological diagnosis (no MASLD, MASLD, MASH). **Lower panel**: sCD14 across fibrosis stages (F0–F3)*.

Plasma sCD14 levels significantly increased across categories at higher fibrosis risk (*p* value for trend test = 0.028). The association between increased sCD14 and MASL/MASH, and, specifically, the presence of linear correlation between sCD14 levels and progressively more severe fibrosis stages was confirmed in the subgroup of individuals undergoing intra-operative liver biopsy during sleeve gastrectomy intervention, performed for clinical indication (Figure 2):

Circulating sCD14 levels showed significant associations with both liver-related and metabolic parameters. At the bivariate correlation analyses, sCD14 was positively associated with the HSI (r = 0.26, *p* = 0.005) and markers of hepatocellular damage (AST: r = 0.28, *p* = 0.002; ALT: r = 0.32, *p* < 0.001); BMI (r = 0.25, *p* < 0.001), waist circumference (r = 0.34, *p* < 0.001), fasting plasma glucose (r = 0.30, *p* = 0.01), and inversely associated with HDL cholesterol (r = −0.23, *p* = 0.016).

Plasma sCD14 levels were significantly higher in female participants (*p* = 0.018) and in presence of obesity (*p* = 0.009), MASLD, as defined by HSI ≥ 36 (*p* = 0.05), and advanced liver fibrosis, defined by FNI ≥ 0.33 (*p* = 0.028), whereas no significant association was observed with the presence of T2D at the univariate regression analysis.

To explore the association between CD14 across all stages of liver fibrosis estimated by FNI, we correlated sCD14 levels with FNI, considered as a continuous variable. Thus, at the linear univariate regression analysis, circulating sCD14 significantly associated with FNI (standardized β = 0.289, *p* = 0.007). This association remained stable after adjustment for age, sex, BMI, waist circumference, CRP, and type 2 diabetes (standardized β = 0.288, *p* = 0.019), indicating that the relationship between sCD14 and fibrosis risk was independent of major metabolic and inflammatory confounders.

Finally, we built a multivariable logistic regression model evaluating determinants of higher liver fibrosis risk in our population, including sex, age, circulating sCD14 levels and traditional metabolic risk factors. After adjustment, circulating sCD14 levels remained independently associated with estimated severe liver fibrosis (OR per 1 SD increase 3.16, 95% CI 1.32–7.55; *p* = 0.010; Table 3).

To further contextualize the performance of sCD14 in relation to established non-invasive fibrosis scores, we performed additional analyses using the FIB-4 index. In our cohort, 79% of individuals were classified as low risk, 19% as intermediate risk, and only 2% as high risk for advanced fibrosis, indicating a limited ability of FIB-4 to capture fibrosis severity in this dysmetabolic population. Consistently, no significant association was observed between circulating sCD14 levels and FIB-4, either when analyzed as a continuous variable (r = 0.076, *p* = 0.478) or across FIB-4 risk categories (1229.8 ± 373.4 vs. 1342.1 ± 378.1 ng/mL, *p* = 0.248).

## 3. Discussion

In this cross-sectional cohort study of dysmetabolic individuals, we investigated the relationship between circulating markers of gut permeability–related immune activation and liver disease severity in MASLD. The main finding is that circulating sCD14 levels were independently associated with liver fibrosis in MASLD. Importantly, this association remained significant after adjustment for major metabolic confounders, supporting the concept that gut-derived innate immune activation may contribute to fibrogenesis beyond traditional metabolic risk factors.

Our results are consistent with, but also extend, previous evidence from Japanese cohorts evaluating sCD14 in NAFLD/NASH. Ogawa et al. [19] demonstrated that sCD14 correlates with histological liver inflammation and discriminates NASH from non-NASH disease, while Nakamura et al. [20] reported associations with ballooning, inflammation, and fibrosis. However, these studies primarily focused on steatohepatitis and inflammatory activity rather than fibrosis risk per se, and the potential confounding effect of obesity, central adiposity, and type 2 diabetes was not systematically addressed.

In contrast, the present study specifically evaluated the relationship between sCD14 and fibrosis risk in a metabolically complex MASLD population while rigorously adjusting for key metabolic variables. Notably, the association between sCD14 and advanced fibrosis remained independent of BMI, waist circumference, and T2D. In addition, unlike prior biopsy-enriched cohorts, our analysis reflects a real-world dysmetabolic population and is supported by histological validation in a bariatric subgroup. Together, these features strengthen the external validity of our findings and suggest that sCD14 may capture fibrogenic risk across the MASLD spectrum rather than merely reflecting steatohepatitis.

Unlike sCD14, LBP levels did not reflect increased fibrosis risk. The divergent behavior of sCD14 and LBP provides further pathophysiological insight. While LBP mainly reflects circulating endotoxin exposure [21], sCD14 mirrors the host immune response to microbial translocation. In our cohort, LBP and the LBP/sCD14 ratio were associated predominantly with steatosis, whereas only sCD14 showed a robust and independent relationship with fibrosis severity. This pattern supports the hypothesis that host immune activation—rather than endotoxin burden alone—may play a more direct role in fibrogenesis within the gut–liver axis. This interpretation is biologically plausible given the established role of CD14–TLR4 signaling in hepatic stellate cell activation and extracellular matrix deposition.

Interestingly, additional analyses using FIB-4 did not show any significant association with sCD14, likely reflecting the limited sensitivity of traditional fibrosis scores in detecting early or intermediate stages of disease in dysmetabolic populations.

The association between sCD14 and fibrosis was corroborated in the histological subgroup, where circulating levels increased progressively across fibrosis stages. Although the biopsy sample was relatively small, the concordance between non-invasive and histological findings strengthens the reliability of the observed relationship. Moreover, the absence of independent associations for broader inflammatory markers such as CRP and TNF-α suggests that sCD14 may reflect a more specific innate immune pathway linked to microbial sensing rather than systemic inflammation per se.

These findings should be viewed within the current conceptual framework of MASLD pathogenesis. While metabolic dysfunction remains the primary driver of hepatic steatosis, accumulating evidence supports a multiple-hit model in which gut barrier impairment, microbial translocation, and innate immune activation amplify liver injury and fibrotic remodeling. In this context, sCD14 may represent an integrated biomarker capturing the interplay between intestinal permeability, immune activation, and hepatic fibrogenesis.

Some limitations should be acknowledged. The cross-sectional design precludes causal inference and does not allow assessment of the prognostic value of sCD14 for fibrosis progression. The histological subgroup was relatively small and limited to bariatric surgery candidates, which may affect generalizability. In addition, circulating endotoxin and gut microbiota composition were not directly assessed, limiting mechanistic interpretation. Moreover, a more extensive characterization of the cytokine profile, particularly of interleukin-related pathways, could have provided further insight into the inflammatory mechanisms underlying these findings and helped refine their biological interpretation. While such analyses were not available in the present cohort, this aspect deserves further investigation in future studies. Finally, residual confounding from unmeasured factors, including diet, medications, and low-grade infections, cannot be completely excluded.

From a clinical standpoint, the identification of non-invasive biomarkers able to stratify fibrosis risk in MASLD remains a major unmet need. If confirmed in longitudinal studies, sCD14 could contribute to multimarker strategies aimed at identifying patients in whom gut-derived immune activation is a relevant driver of disease progression. These data also raise the possibility that therapeutic interventions targeting intestinal permeability, microbial translocation, or CD14–TLR4 signaling could have antifibrotic potential in selected MASLD phenotypes.

Future studies should determine whether sCD14 predicts incident fibrosis progression and liver-related outcomes over time. Integration with microbiome profiling, direct endotoxin measurements, and functional assessment of intestinal barrier integrity will be essential to clarify causality. Validation in larger and more diverse MASLD populations is also warranted to define the clinical utility and optimal thresholds of sCD14. Finally, interventional studies evaluating whether modulation of the gut–liver axis reduces sCD14 levels and attenuates fibrogenesis represent an important next step.

## 4. Materials and Methods

### 4.1. Study Design and Population

This observational cross-sectional study was conducted at the Diabetes Outpatient Clinic of Sapienza University of Rome, Italy. Adult subjects consecutively referring to the clinic for metabolic evaluation and management of T2D were screened for inclusion.

Eligible participants were men and women aged ≥18 years with available complete clinical, anthropometric, and biochemical data. Exclusion criteria were: excessive alcohol consumption, defined as a daily intake >30 g in men and >20 g in women; positivity for hepatitis B surface antigen or hepatitis C virus antibodies; history or clinical evidence of chronic liver diseases other than MASLD, including autoimmune hepatitis, hemochromatosis, Wilson’s disease, or cholestatic liver disorders; previous diagnosis of liver cirrhosis; and current or past use of medications known to induce hepatic steatosis or liver injury, such as systemic corticosteroids, estrogens, methotrexate, amiodarone, tetracyclines, or calcium channel blockers. A total of 139 subjects fulfilling all inclusion and exclusion criteria were included in the final analysis.

All participants underwent a routine clinical assessment; body weight was measured with subjects wearing light clothing and no shoes, and height was measured to the nearest 0.5 cm. The body mass index (BMI) was calculated as weight divided by height squared (kg/m^2^). Waist circumference was measured using a non-elastic tape at the midpoint between the lower margin of the last palpable rib and the iliac crest, with the subject standing and breathing normally. Blood pressure was measured in the seated position after at least 5 min of rest using an automated sphygmomanometer. Three consecutive measurements were obtained, and the mean value was used for analyses.

A detailed medical history was collected by structured interview and review of medical records, including information on smoking status, presence and duration of T2D, complications, comorbidities, and ongoing pharmacological treatments.

Venous blood samples were collected in the morning after an overnight fast of at least 12 h. Plasma glucose, glycated hemoglobin (HbA1c, %—mmol/mol), total cholesterol (mg/dL), high-density lipoprotein (HDL) cholesterol (mg/dL), low-density lipoprotein (LDL) cholesterol (mg/dL), triglycerides (mg/dL), alanine aminotransferase (ALT, IU/L), and aspartate aminotransferase (AST, IU/L), gamma glutamyl transpeptidase (IU/L), serum creatinine (mg/dL), were measured using standard enzymatic colorimetric methods.

Fasting serum insulin was determined by immunoassay, and insulin resistance was estimated using the Homeostasis Model Assessment of Insulin Resistance (HOMA-IR). Renal function was assessed by measuring serum creatinine and calculating the estimated glomerular filtration rate (eGFR) using the CKD-EPI equation. Type 2 diabetes was diagnosed according to current international diagnostic criteria or documented use of glucose-lowering medications.

### 4.2. Liver Assessments

Hepatic steatosis was estimated in all the study participants using the Hepatic Steatosis Index (HSI), a validated surrogate marker which includes aminotransferase levels, BMI, sex, and diabetes status [22]. MASLD was diagnosed based on the presence of hepatic steatosis in association with metabolic dysfunction, in accordance with current international consensus definitions [23]. Liver fibrosis risk was estimated using the Fibrotic NASH Index (FNI), an age-independent, non-invasive, validated score derived from routinely available biochemical parameters, such as AST, HbA1c, and HDL cholesterol [24]. The FNI was calculated for each participant, and liver fibrosis severity was categorized according to established cut-offs; advanced liver fibrosis was defined by an FNI value > 0.33 [24]. In a subgroup of subjects undergoing bariatric surgery for clinical indications, intraoperative liver biopsy specimens were available. Histological assessment was performed by an experienced pathologist blinded to clinical and laboratory data. Liver specimens were evaluated using both the NAFLD Activity Score (NAS) and the Steatosis–Activity–Fibrosis (SAF) scoring system to assess steatosis, inflammatory activity, and fibrosis stage [25,26].

### 4.3. Assessment of Gut Permeability and Immune Activation Markers

Intestinal permeability, endotoxemia, and immune activation were estimated by measuring plasma levels of soluble CD14 (sCD14), and lipopolysaccharide-binding protein (LBP) by ELISA kits (Human CD14 ELISA Kit, R&D System, Minneapolis, USA) and then the LBP/sCD14 ratio was calculated. As additional circulating markers of inflammation/immune activation in metabolic disease, plasma TNF-a, and zonulin concentrations were determined using MILLIPLEX Map Human Cardiovascular Disease (CVD) Magnetic Bead Panel 3 (Acute Phase) (HCVD3MAG-67K, Merck KGaA, Darmstadt, Germany) on a Luminex-200 System. Plasma samples were stored at −80 °C until analysis. All samples were analyzed in duplicate, and mean values were used for statistical analyses. Intra- and inter-assay coefficients of variation were within acceptable limits as specified by the manufacturers.

### 4.4. Statistical Analysis

Statistical analyses were performed using SPSS software version 27.0 (IBM Corp., Armonk, NY, USA). Continuous variables are reported as mean ± standard deviation or median (interquartile range), according to data distribution. Categorical variables are expressed as frequencies and percentages. Associations between markers of gut permeability and indices of liver steatosis and fibrosis were evaluated using Pearson or Spearman correlation analyses, as appropriate. Differences between groups were assessed using the Student’s *t*-test or Mann–Whitney U test for continuous variables and the chi-square test for categorical variables. Logistic regression analyses were performed to identify independent predictors of advanced liver fibrosis. Multivariable models were constructed including clinically relevant covariates and potential confounders, such as age, sex, BMI, waist circumference, and presence of T2D. Results are reported as odds ratios (ORs) with 95% confidence intervals (CIs). A two-sided *p*-value < 0.05 was considered statistically significant.

## 5. Conclusions

Circulating sCD14 shows potential as a clinically relevant biomarker of fibrogenic risk in MASLD, capturing aspects of gut–immune–liver interplay not fully explained by metabolic burden alone. If confirmed in longitudinal and interventional studies, sCD14 may contribute to improved risk stratification and help identify patient subgroups who could benefit from intervention targeting the gut–liver axis.

## Figures and Tables

**Table 1:** Clinical characteristics of the study population.

	Mean ± SD
Age (years)	48.8 ± 10.9
Female (%)	56.8% *
BMI (kg/m^2^)	33.7 ± 9.5
Waist circumference (cm)	110.9 ± 18.6
Systolic BP (mmHg)	128.9 ± 14.5
Diastolic BP (mmHg)	80.9 ± 8.8
Total cholesterol (mg/dL)	187.7 ± 37.6
HDL cholesterol (mg/dL)	49.7 ± 15.4
LDL cholesterol (mg/dL)	108.2 ± 34.6
Triglycerides (mg/dL)	149.2 ± 83.3
Fasting glucose (mg/dL)	116.1 ± 37.1
HbA1c (%)	6.2 ± 1.0
AST (U/L)	27.7 ± 15.2
ALT (U/L)	35.4 ± 25.3
GGT (U/L)	35.8 ± 39.0
Uric acid (mg/dL)	5.7 ± 1.1

Data are presented as mean ± standard deviation (SD) or * percentage, as appropriate.

**Table 2:** Comparison of clinical, metabolic, and biochemical characteristics according to Fibrotic NASH Index (FNI)–defined liver fibrosis risk.

	Low–Intermediate Fibrosis Risk (FNI < 0.33) (N = 47)	High Fibrosis Risk (FNI ≥ 0.33) (N = 92)	*p*-Value
Age (years)	48.12 ± 10.44	46.78 ± 8.26	0.533
Female (%)	58.6%	45.8%	0.278
Diabetes (%)	57%	92%	0.001
Obesity (%)	71%	70%	0.89
BMI (kg/m^2^)	36.10 ± 7.70	35.29 ± 12.05	0.763
Waist circumference (cm)	113.57 ± 15.47	115.83 ± 17.64	0.560
Total cholesterol (mg/dL)	185.17 ± 36.57	183.75 ± 43.69	0.887
HDL (mg/dL)	50.70 ± 13.07	40.21 ± 17.40	0.008
LDL (mg/dL)	104.79 ± 34.12	104.14 ± 36.31	0.939
Triglycerides (mg/dL)	145.37 ± 77.96	188.21 ± 94.37	0.048
Fasting blood glucose (mg/dL)	117.93 ± 40.48	139.13 ± 34.77	0.015
Fasting plasma insulin (mU/mL)	11.74 ± 5.79	10.08 ± 4.35	0.338
HbA1c (%)	5.98 ± 0.87	6.96 ± 1.12	0.0001
AST (IU/L)	22.57 ± 6.20	42.00 ± 20.10	0.0001
ALT (IU/L)	29.23 ± 12.45	53.92 ± 37.84	0.002
GGT (IU/L)	31.65 ± 31.76	63.08 ± 62.88	0.021
Uric acid (mg/dL)	5.80 ± 1.12	5.42 ± 1.18	0.328
C-reactive protein (mg/dL)	2.03 ± 3.10	2.53 ± 2.74	0.467
TNF-α (pg/mL)	2.68 ± 2.02	4.27 ± 2.15	0.184
sCD14 (ng/mL)	1224.7 ± 342.6	1429.4 ± 373.6	0.023
LBP (µL/mL)	28.6 ± 23.7	30.5 ± 34.1	0.788
LBP/CD14	0.03 ± 0.04	0.02 ± 0.01	0.339
Zonulin (mg/dL)	449 ± 1133	267 ± 527	0.536

*Data are expressed as mean ± standard deviation or percentage, as appropriate. p-values refer to between-group comparisons*.

**Table 3:** Multivariable logistic regression analysis of factors associated with estimated severe liver fibrosis. (FNI ≥ 0.33).

	β	S.E.	*p*-Value	OR	95% CI
Age	−0.192	0.358	0.592	0.825	0.409–1.665
Sex (M vs. F)	0.237	0.821	0.772	1.268	0.254–6.337
sCD14	1.150	0.445	0.010	3.158	1.321–7.550
BMI	0.078	0.560	0.889	1.082	0.361–3.241
Waist circumference	0.190	0.603	0.753	1.209	0.371–3.938
CRP	−0.167	0.297	0.574	0.846	0.473–1.514
Diabetes (Yes vs. No)	3.135	1.089	0.004	22.998	2.722–194.329

*Results are presented as β coefficients, standard errors (S.E.), odds ratios (OR), and 95% confidence intervals (CI). Continuous variables were standardized before inclusion in the model. ORs represent the change in odds of estimated severe liver fibrosis per 1 standard deviation increase in the corresponding variable. Categorical variables are presented as indicated*.

## Data Availability

The data presented in this study are available from the corresponding author upon request. The data are not publicly available due to privacy restrictions and lack of specific patient consent.
